# Evaluation of a Novel Non-Destructive Catch and Release Technology for Harvesting Autologous Adult Stem Cells

**DOI:** 10.1371/journal.pone.0053933

**Published:** 2013-01-22

**Authors:** Nicholas Bryan, Fiona C. Lewis, Damian Bond, Christopher Stanley, John A. Hunt

**Affiliations:** 1 Clinical Engineering, United Kingdom Centre for Tissue Engineering, Institute of Ageing and Chronic Disease, University of Liverpool, Liverpool, United Kingdom; 2 CellCap Technologies Ltd., The Greens Farm, Todmorden, Bacup, United Kingdom; Instituto Butantan, Brazil

## Abstract

**Background:**

Cell based therapies are required now to meet the critical care needs of paediatrics and healthy ageing in an increasingly long-lived human population. Repair of compromised tissue by supporting autologous regeneration is a life changing objective uniting the fields of medical science and engineering. Adipose stem cells (adSCs) are a compelling candidate for use in cell based medicine due to their plasticity and residence in numerous tissues. Adipose found in all animals contains a relatively high concentration of stem cells and is easily isolated by a minimally invasive clinical intervention; such as liposuction.

**Methods:**

This study utilised primary rat adipose to validate a novel strategy for selecting adult stem cells. Experiments explored the use of large, very dense cell-specific antibody loaded isolation beads (diameter 5x–10x greater than target cells) which overcome the problem of endocytosis and have proved to be very effective in cell isolation from minimally processed primary tissue. The technique also benefited from pH mediated release, which enabled elution of captured cells using a simple pH shift.

**Results:**

Large beads successfully captured and released adSCs from rat adipose, which were characterised using a combination of microscopy, flow cytometry and PCR. The resultant purified cell population retains minimal capture artefact facilitating autologous reperfusion or application in *in vitro* models.

**Conclusion:**

Although evidenced here for adSCs, this approach provides a technological advance at a platform level; whereby it can be applied to isolate any cell population for which there is a characterised surface antigen.

## Introduction

Stem cell niches exist within almost all tissues of an adult organism; their function to specifically localise and differentiate into a specific type of cell to renew and repair the tissue in which they reside has been realised scientifically [1], [2]. However, a fundamental cellular and biochemical understanding of the precise mechanisms behind their physiological functions are yet to be defined, and therefore hampers our ability to harness their potential in efficacious and cost effective medicine [3]. Stem cells have been successfully isolated from a diverse range of tissues, including bone marrow [4]–[6], pancreas [7], adipose [8], [6], dental pulp [9]–[11] and umbilical tissues [12]–[13] and their multilineage potential demonstrated through directed differentiation and functionalisation into representatives from all three developmental germ layers; a characteristic historically reserved solely for stem cells of embryonic origin [14]–[16].

Extracting stem cells from their associated tissue in a manner which renders them viable, phenotypically stable and suitable for therapeutic application has presented a major challenge to the field of cell biology but offers a tantalising omnipotent cell source for regenerative medicine [17]. When considering sources of stem cells, lipoaspirate presents itself as a favourable, readily accessible supply, which can be obtained through minimally invasive procedures, without donor site morbidity [18]–[19]. Additionally, the concentration of stem cells within adipose has been reported to be significantly higher than bone marrow [20]. Coupled with the large quantities of lipoaspirate that can be harvested at any one time, adipose may be considered as a future gold standard stem cell source. Immunophenotyping of cultured adSCs has also revealed >90% similarity with bone marrow-derived stem cells including CD90, CD29, CD44, CD73 and CD105 cell surface antigens [20]–[21].

Isolation of stromal vascular fraction (SVF) from rat adipose was first achieved by Rodbell *et al*. in the 1960 s. Despite this, the notion of adSCs was not widely recognised until 2001 when Zuk *et al*. demonstrated SVF contained large numbers of adSCs, which could differentiate into osteogenic, chondrogenic, adipogenic and myogenic lineages [22]. To date, the majority of approaches for isolating adSCs from SVF are based on the same basic principle; exploiting the ability of adSCs to adhere to plasma treated tissue culture polystyrene substrates. However, this simple but crude approach leads to a heterogeneous culture that contains a variety of adherent cells which includes fibroblasts and endothelial cells, ultimately resulting in a population of which adSCs are a minority [23].

The phenotypic, functional and particularly immunomodulatory effects of prolonged adSC *in vitro* culture are not fully understood, therefore robust and reproducible characterisation of freshly isolated adSCs would present a breakthrough in interpreting complex adSC cell biology. However this has largely been hindered by their rarity and the ability to isolate substantial numbers from fresh tissue to perform immediate and reproducible molecular biology. Several methods are available to isolate adSCs and other primary cells. Currently the two most commonly applied techniques are cell sorting by flow cytometry and paramagnetic particle isolation, both of which allow selection of cells based on antibody/antigen immunolabelling. Flow cytometry utilises fluidic processing to localise target cells into drops or diverted pathways. There are however significant hydrodynamic forces associated with this, which stem cells in particular are affected by. Magnetic particles currently in use are 50 nm−4.5 µm diameter, to which cell-specific antibodies are attached. These bind cells, which then become decorated with the particles; the complexes are subsequently exposed to a magnetic field resulting in separation of specific tagged cells from a heterogeneous cell population. This provides a convenient method of selecting cells; however the very small size of the paramagnetic particles means they are typically internalised into the cell, resulting in potential phenotypic changes [23]. Additionally, these small particles are not compatible with the dense proteinaceous matrix of primary tissue where they are observed to bind strongly to tissue materials and even air bubbles (unpublished observations): therefore extensive tissue pre-processing to create a simpler matrix for cell capture is required.

Commonly flow cytometry or immunomagnetic selection relies on negative depletion to remove non-stem cells from the culture milieu. This technique leaves stem cells without phenotypic compromise by selection chemistry; however the heterogeneity of primary tissue renders it impossible to attain homogeneous stem cell cultures even after several rounds of depletion [24]–[25]. Positive selection of stem cells is possible, however due to the nature of flow cytometry both positive and negative populations of cells will subject to the physical forces of flow cytometric sorting. In the case of paramagnetic bead isolation, the positive cell fraction runs the risk of endocytosis of small magnetic particles, which may render them transcriptionally modified. The derivation of pure stem cell populations for *in vitro* characterisation, pre-clinical research and ultimately translation into regenerative medical therapeutics remains a rate limiting challenge to the progression of stem cell medicine and biology.

Here we provide experimental evidence for a novel cell isolation system for high affinity catch and release of adSCs from minimally processed adult tissue. This system utilises large, dense separation beads populated with an antibody binding ligand. The ligand binds cell-specific antibody in a pH dependent manner permitting simple cell release with a small shift in reaction pH. Herein this system was utilised to isolate and release adSCs from rat adipose SVF.

## Materials and Methods

### Ethics statement

All studies adhered to UK home office use of animals in scientific procedures guidelines and were approved by the Institutional Review Board of the University of Liverpool.

### Isolation of stromal vascular fraction (SVF) from rat adipose tissue

Subcutaneous and visceral adipose were dissected from adult Wister rats. Primary tissue was washed 3x using PBS, coarsely macerated using sterile dissection scissors and liquidised by forcing through a 10 ml syringe. Digestion was achieved by incubation in 0.2% collagenase/PBS (Sigma-Aldrich, UK) (37°C, 90 mins, 50% v/v collagenase solution/tissue homogenate). After this time had elapsed the reaction was neutralised by addition of 10% fetal calf serum. The digest was passed through a 100 µm cell strainer then centrifuged (400 g, 10 mins). To remove residual erythrocytes, cells were suspended in 200 µl PBS with 1 ml Optilyse C (Beckman Coulter, RT, 10 mins). 10 ml PBS was then added to the erythrolysed cell suspension before a final centrifugation to recover SVF cells (400 g 10 mins). Resulting cells were suspended in an appropriate volume of PBS and numerated using a hemocytometer.

### Immunofluorescent staining and FACS analysis

SVF was labelled with FITC conjugated mouse anti rat CD90, CD29, CD44, CD45, and CD31 (15 mins, 4°C, 1 µg antibody/10^5^ cells). A FITC conjugated isotype control (IgG_1_) was used at the same concentration to allow post-hoc subtraction of non-antigen-specific fluorescence. The percentage cells in the SVF fraction expressing these antigens was quantified using flow cytometry to numerate cells with associated antibody mediated fluorescence.

### CD90^+^ isolation: protein A-coated beads (non-reversible antibody binding)

CD90^+^ cell capture was achieved by labelling cells and loading Protein A-coated capture beads (50–200 µm diameter, CellCap Technologies Ltd) with CD90 antibody at the following concentrations: 1 µg antibody/10^5^ cells and 1 µg antibody/10 µL beads. Equal volumes of cell suspensions and beads were incubated in a final volume of 1 ml PBS with gentle rolling in 1.5 ml polypropylene tubes on a roller table (30 mins, 4°C). Reactions in which neither cells nor beads received antibody were performed as a negative control.

Post bead/cell interaction, the percentage of cells specifically depleted by specific capture was quantified using flow cytometry, again based on cellular events associated with antibody mediated FITC fluorescence.

### RNA isolation

The following solutions were prepared prior to RNA isolation (all reagents Qiagen, UK unless stated otherwise). 44 ml of ACS grade 100% ethanol was added to 6 ml wash buffer (RPE), while 10 µl of 1 M β-mercaptoethanol (Sigma-Aldrich UK) was added to 1 ml lysis buffer (RLT).

Prior to RNA extraction cells were washed with PBS (3×5 minutes, room temperature). Following this, 350 µl of buffer RLT was added to each sample and incubated for 5 minutes at room temperature. Resulting lysates were transferred to QIAshredder columns and spun at 13400 g for 2 minutes. 250 µl of 100% ethanol was added to the elutant, transferred to an RNeasy mini column and spun at 13400 g for 15 seconds. 500 µl wash buffer (RW1) was then added to the column and incubated for 5 minutes at room temperature before being spun at 13400 g for 15 seconds. Following this, 500 µl pre-warmed buffer RPE was added to the column and spun for 13400 g for 15 seconds. This step was repeated a second time and spun at 13400 g for 2 minutes. Finally, to elute RNA columns were transferred to RNase free tubes and 30 µl RNase free ddH_2_O added, incubated at room temperature for 2 minutes then spun at 13400 g for 1 minute. Quantity and purity of RNA was determined by spectrophotometry (260/280 nm absorbance). Only samples that had a 260/280 nm absorbance between 1.9 and 2.1 were used in subsequent experiments.

### Real-time PCR (qRT-PCR)

Prior to reverse transcription the following stock solutions were created. Stock 1∶1 µl 50 µM Oligo(dt)_20_, 1 µl 10 mM dNTP cocktail, 9 µl RNAse free ddH_2_O. Stock 2∶4 µl 5x first strand buffers, 1 µl 0.1 M DTT, 1 µl RNaseOUT recombinant RNAse inhibitor (40 U/µL) and 1 µl superscript III RT (200 U/µl). The above stock solutions were suitable quantities for the reverse transcription of 2 µl of RNA (15–200 µg/ml RNA).

2 µl of RNA was added to stock 1 denatured at 65°C for 5 minutes followed immediately by a 1 minute chill at −20°C. Stock 2 was then added, heated for 40 minutes at 50°C, followed by a further 15 minutes at 70°C. All reagents were purchased from Invitrogen, UK.

qRT-PCR reactions were assembled containing 2 µl cDNA diluted 100 fold using molecular biology grade ddH_2_O, 0.5 µl sense primer (100 µM), 0.5 µl antisense primer (100 µM), 7.5 µl Sybr green single tube PCR master mix (Bio-Rad, UK) and 4.5 µl molecular biology grade ddH_2_O.

Primers for gene of interest (CD90) and reference gene (β-actin) were designed in house. (CD90 sense: CTGCTGAGCCTTTGTGGAC, anti-sense; GCATCTTTATTGAGTGTG, T_A:_ 50.1°C. β-actin: sense; GGGACCTGACTGACTACCTC, anti-sense; GCCATCTCTTGCTCGAAG, T_A:_ 53.9°C).

Reactions were denatured for 3 minutes at 95°C then cycled 40 times at 95°C for 30 seconds, followed by 40 cycles of annealing; 55°C for 30 seconds, 95°C for 30 seconds and finally 40 cycles at 55°C for 10 seconds. For all qRT-PCR reactions n = 3/sample. Using inter-experimental variations CD90 transcript could be calculated by normalisation to the ubiquitously expressed β-actin reference gene based on standard threshold cycle (C_T_) analysis: 2^–(ΔCTsample – ΔCTcontrol)^ where ΔC_T_  =  C_T_ gene of interest-C_T_ reference gene.

### Primer design

Coding strand cDNA sequences (CDS) of genes of interest were identified using the genome search platform www.ncbi.nlm.nih.gov. The CDS sequence was copied into the primer design platform, Beacon Designer V.7.21 (Premier Biosoft International, USA). Amplicons of 75–200 bp were selected for optimal compliance with SYBR green chemistry, along with low guanine-cytosine (GC) content and an annealing temperature of 55.0+/−5.0°C. In addition, all primers were designed between 18–24 bp in length. The proposed primers were verified for tertiary structures using the DNA mfold server provided by M. Zuker at http://frontend.bioinfo.rpi.edu/applications/mfold/cgi-bin/dna-form1.cgi. Primers that formed complex hairpin loops at the annealing temperature identified by the primer design platform were discarded as it was unlikely that they would anneal correctly.

Cross sequence homology was investigated by perfoming a basic local alignment search (BLAST) was then used to interrogate the rat genome to identify regions complementary to the designed primers outside of the target gene. Primers displaying any cross sequence homology were rejected.

Primers were synthesised at a production scale of 25 nM by Invitrogen.

### CD90^+^ isolation: mixed-mode ligand-coated beads (reversible antibody binding)

In order to optimise loading of CD90 FITC-conjugated antibodies on mixed-mode (i.e. containing both aromatic and acidic groups) ligand beads (50–200 µm diameter, supplied by CellCap Technologies Ltd) several buffer configurations were explored; 200 mM TRIS or 0.1 M phosphate buffer adjusted to pH 5, 6 or 7.4. In each case beads were washed 3x in buffer before addition of 1 µg antibody/1 ml beads (15 mins, 4°C). Beads were then washed three times in the corresponding buffer to remove unbound antibody and antibody loading confirmed using fluorescent microscopy. Release was achieved by incubating beads for 15 mins at 4°C, at either pH 7.4 or 8.4, an additional blocking variable was also added which included incubation with 10% rabbit serum for 15 min prior to transfer to release buffer.

## Results

### Rat SVF characterisation and capture antigen selection

**Figure 1:**
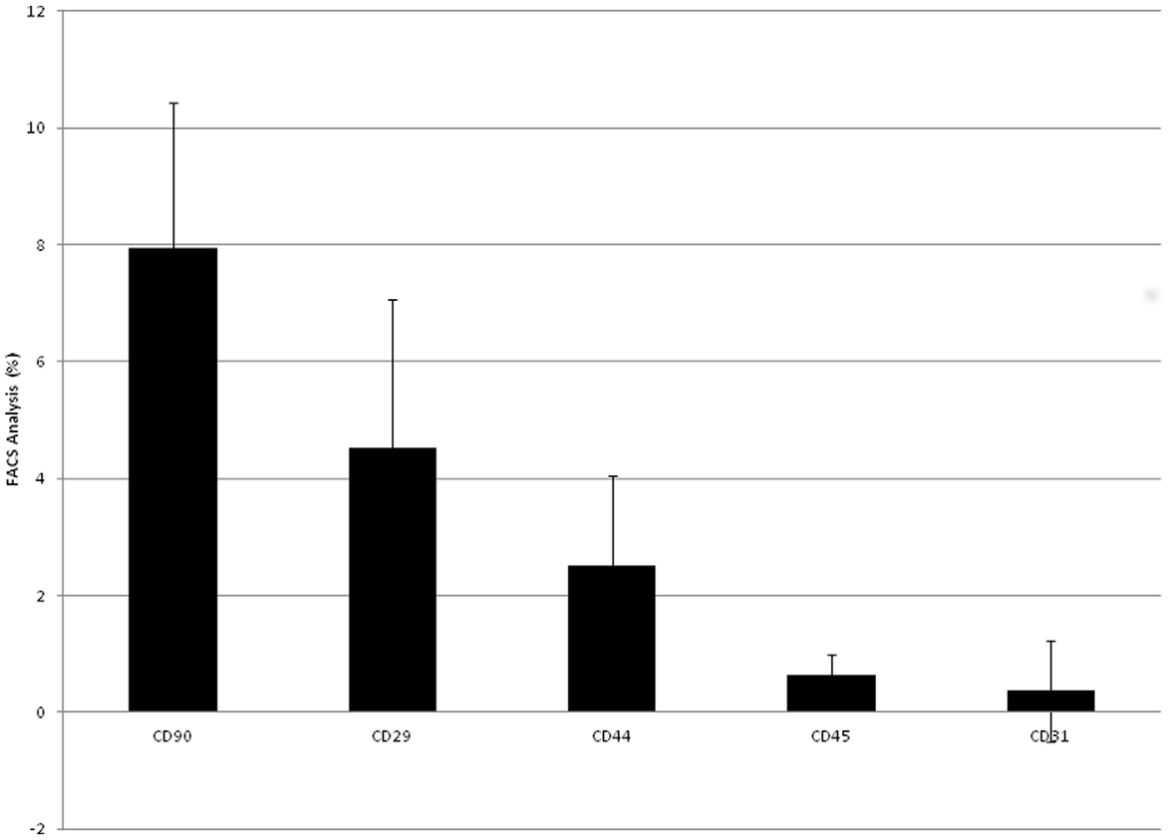
Flow cytometric characterisation of rat primary adipose. A: SVF Characterisation. Error bars represent 1 standard deviation from the mean, n = 7 (inter-animal repeats). B: Percentage CD90+ cells present in subcutaneous and intra-abdominal adipose tissue. Error bars represent 1 standard deviation from the mean, n = 3 (inter-animal repeats).

Prior to cell isolation SVF was characterised using a panel of surface antigens (CD90, CD29, CD44, CD45 and CD31) which validated CD90^+^ as the most appropriate target for adSC isolation from this tissue. Flow cytometry allowed conclusion that of the antigens analysed, CD90 was the most abundant ranging between 5–10% of total SVF cells, n = 7 (inter-animal repeats) (Fig. 1). It was also found that the percentage of CD90^+^ adSCs in SVF did not vary as a function of anatomical origin of the source material, with inter-abdominal and subcutaneous adipose yielding identical adSC (CD90^+^) concentrations.

### CD90^+^ isolation: protein A-coated beads (non-reversible antibody binding)

**Figure 2:**
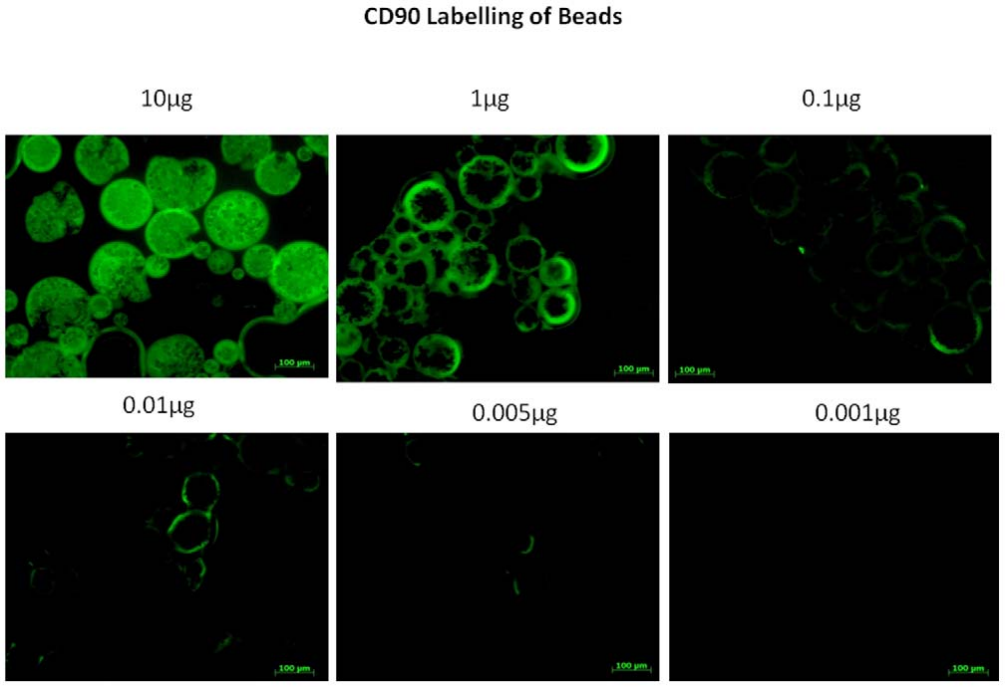
Fluorescent microscopic observation of successful CD90 FITC antibody loading of protein A beads.

Initial experiments utilised protein A-coated beads (50–200 µm) to deplete CD90^+^ cells from SVF. Before cell isolation, loading of CD90 antibody onto beads was confirmed by exploiting the antibodies FITC conjugation to visualise antibodies co-localised with beads using fluorescent microscopy (Fig. 2).

**Figure 3:**
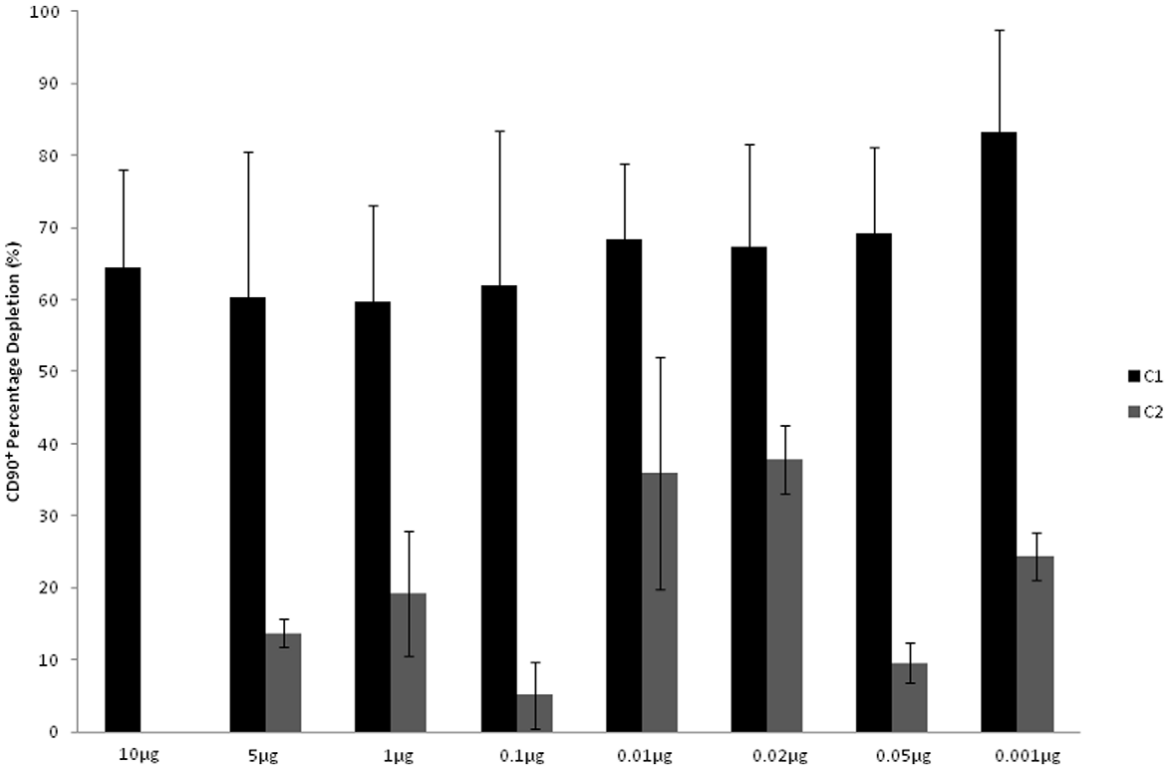
Percentage CD90+ cell depletion as a function of decreasing antibody concentration on capture beads. Antibody concentration on the cells remained constant; 1 µg antibody/10^5^ cells. X; bead antibody concentration, Y: percentage CD90 cell depletion. Error bars represent 1 standard deviation, n = 5 (technical replicates). C1 – both cells and beads labelled, C2 – beads alone labeled.

**Figure 4:**
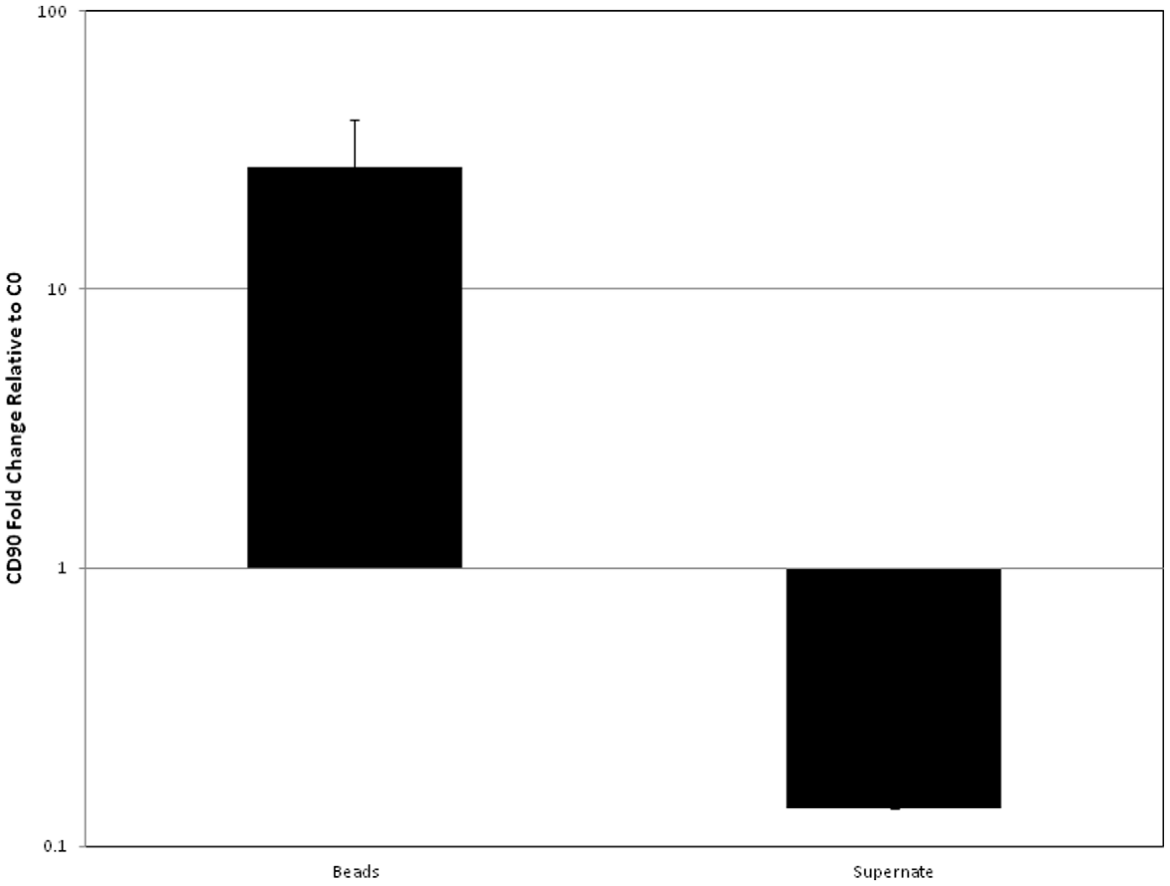
qRT-PCR analysis of CD90 fold change relative to a control capture containing no antibody after normalisation to a beta-actin control. Error bars represent 1 standard deviation from the mean, n = 3 (technical replicates).

Flow cytometric analysis after cell/bead interaction concluded that labelling/loading of both cells and beads with antibody provided the greatest CD90^+^ depletion and that the antibody concentration used to load the beads could be considerably reduced without compromising CD90^+^ cell depletion. A mean depletion of 80% was recorded when cells were pre-labelled with 1 µg antibody/10^5^ cells and beads loaded with a very low antibody concentration; 0.001 µg (Fig. 3). To further confirm CD90^+^ capture, RNA was isolated from both components of the capture reaction; beads and surrounding capture supernatents, and qRT-PCR performed to compare CD90 presence relative to a negative control capture in which no antibody was added to the system. This demonstrated that cells bound to the bead surface expressed CD90 while the contrary was true of the cells which remained unassociated with beads in the surrounding reaction supernatant. This showed that CD90+ cells in the reaction mixture were associated with the bead surface post CD90 targeted cell/bead interaction (Fig. 4).

### CD90^+^ isolation: mixed-mode ligand-coated beads (reversible antibody binding)

**Figure 5:**
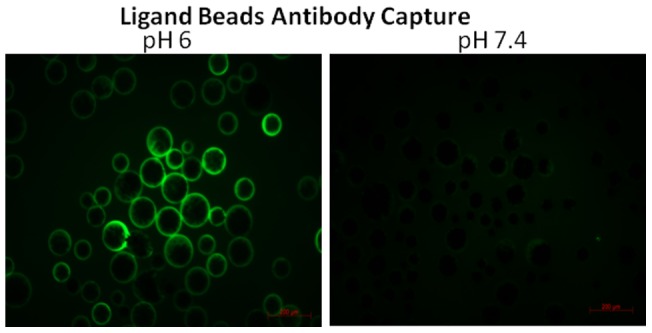
Optimisation of appropriate reaction conditions for cell capture using ligand beads using fluorescent microscopy to observe ligand particles co-localised with FITC conjugated antibody across a pH gradient.

To evaluate this bead coating it was necessary to replicate loading of FITC-conjugated CD90 antibody to the surface of mixed-mode ligand coated beads. These beads are decorated with an aromatic acid ligand which has the ability to bind and release Ig in a pH-mediated manner. This suggested them as ideal candidates for isolation and recovery of cells using antibody reliant mechanisms [26]. Briefly, antibody loading was investigated using both TRIS and phosphate buffers at pHs 5, 6 and 7.4 with Fluorescent microscopy to observe FITC Ig/bead co-localisation. pHs 5 and 6 facilitated successful antibody loading whilst pH 7.4 did not (Fig. 5). Release of bound antibody was interrogated using both TRIS and phosphate buffers at pHs 7.4 and 8.4+/– a pre-release incubation with 10% rabbit serum. optimal release (leaving no visualisable FITC fluorescence) was observed after pre-incubating antibody-coated beads with rabbit serum before transfer to TRIS pH 8.4. This mechanism was found to be instantaneous.

**Figure 6:**
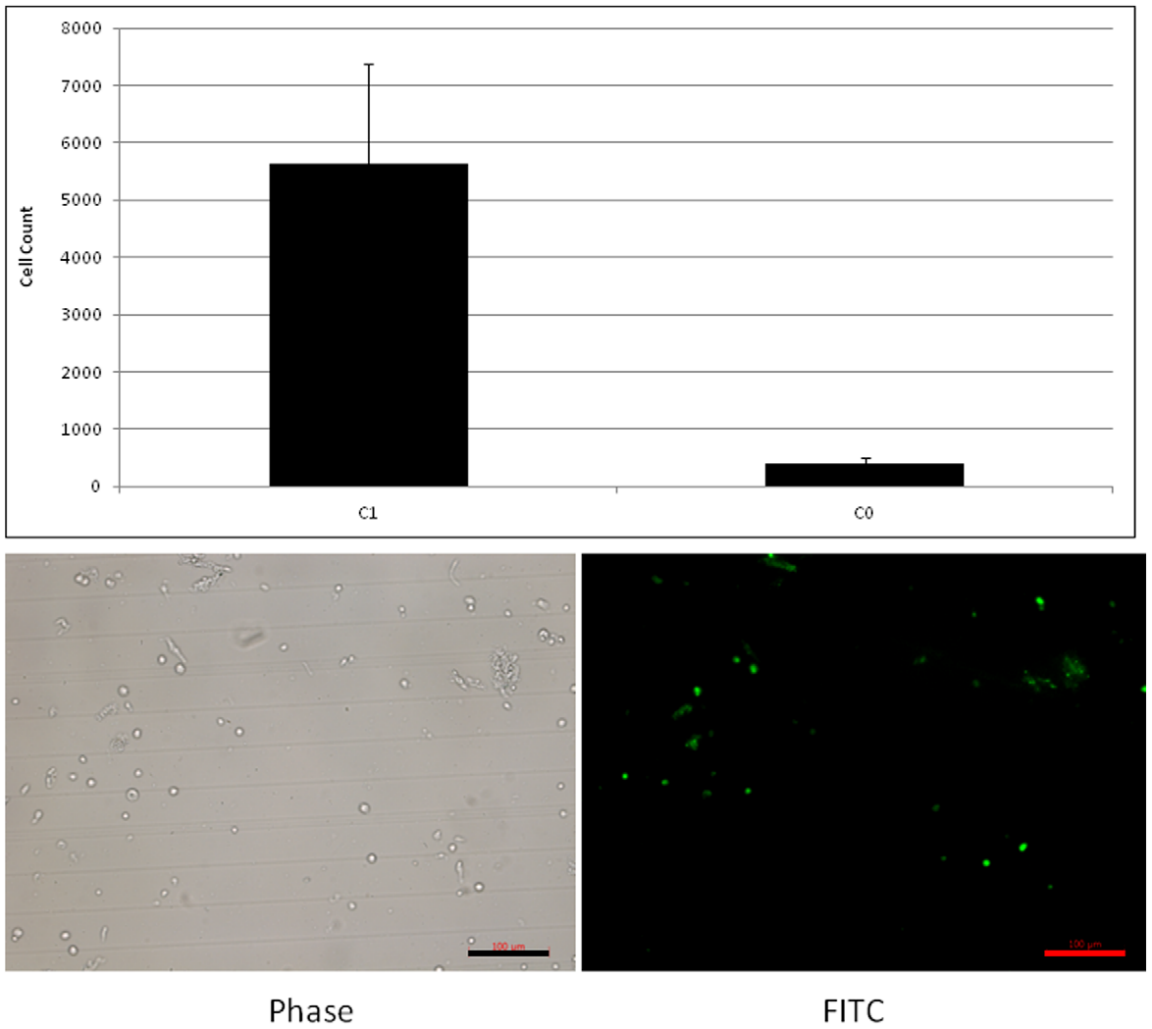
A: Quantification of released cells using a hemocytometer. Error bars represent 1 standard deviation from the mean, n = 4 (technical replicates) and fluorescent microscopic characterisation of the released cell fraction by observation of CD90 FITC labeling. scale bar represents 100 µm.

Ligand beads loaded with either 10 µg or 0.001 µg/ml CD90 antibody were combined with CD90 labelled SVF (1 µg antibody/10^5^ cells) in 200 mM TRIS, pH 5. Following incubation, supernatants were subject to flow cytometric analysis, which revealed depletion of CD90^+^ cells to an extent comparable to their Protein A-coated equivalent. Cell laden beads were incubated with rabbit serum for 15 minutes at 4°C before washing in 200 mM TRIS pH 8.4 to elute bound cells. The beads were allowed to settle, supernatant removed and centrifuged for 10 minutes at 1500 rpm. The mean number of recovered cells was 5.6×10^3^, which correlated with the initial population of 5.0×10^3^ CD90^+^ cells/10^5^ cells, n = 4 (Fig. 6). Post-elution the identity of released cells was confirmed by fluorescent microscopic visualisation of CD90 FITC conjugated antibody associated with the cell surface (Fig. 6).

## Discussion

adSCs present an untapped source of pluripotent cells for future medicine. However their isolation and characterisation requires significant advancement to enable their potential to be realised. An ideal isolation procedure would facilitate straight forward clinical translation from a regulatory perspective whilst also fundamentally increasing first principles biological understanding of the role of adSCs in normal tissue homeostasis, ageing, wound healing and chronic disease.

Development of a cell isolation system that provides high purity and yield whilst avoiding receptor mediated endocytosis of small immunomagnetic particles and fluidic shear and extreme hydrodynamic forces of flow cytometry will provide a superior and urgently required alternative to today's widely applied selection methods. Ideally, an advanced new approach should provide a homogeneous population without compromising the immunoprivilege of autologous cells. The system should perform in complex, matrix rich primary tissues with minimal preparatory pre-processing, which can induce cellular transcriptomic and secretomic modifications.

Immunophenotyping has identified a number of adSC surface molecules, which provide targets to exhume these cells from the heterogeneous milieu of stromal tissue. We have evidenced using a relevant animal model that CD90 is the most appropriate molecule for isolating adSCs. In this rat model CD90 expression was relatively low ranging between 5–10% compared to previous reports investigating human adipose adSCs, where approximately 50% of cells are CD90^+^ [27]–[28]. Nevertheless CD90^+^ cells were repeatedly identified in the SVF of rat adipose tissue from both the intra and extra-abdominal regions, which is highly clinically relevant, demonstrating minimally invasive subcutaneous adipose (lipoaspirate) is no less potent a source of stem cells than visceral adipose. This reduces the potential for donor site morbidity during tissue harvesting associated with considerably more invasive obtaining of ASCs by bone marrow aspiration.

In this study we used a preparation of large, dense antibody-loaded beads with diameters ranging from 50–200 µm (manufacturer's specification) as the core of the cell-isolation system. Previously the use of beads in this size range has not been considered favourable for isolation of mammalian cells due to an expectation that binding kinetics would be too slow [29], the cell loading capacity of the beads would be too low due to a lack of surface area [29] or there would simply be a lack of interaction between cells and beads of this size [30]. A previous report on an end-over-end mixed batch adsorption process using large beads (mean diameter 61 µm) did not perform well and this was attributed to poor suspension of the beads, a lack of contact of cells with the beads and the possibility of mechanical disruption of the cells on the bead surface [31]–[32]. In contrast to these earlier reports we have shown that the use of large beads in a ‘roller bottle’ format has proved to be a very effective method of cell capture. We attribute this to the ease with which the large number of high density beads move through SVF without mechanical restriction, the repeated sedimentation and resupension cycles that they go through and the very high affinity of the interaction when both beads and cell surfaces are populated with antibody. This implies 2 levels of interaction during capture; the antibody on the beads interacting with cell surface antigen in addition to the antibody on the cells binding the Ig specific ligand on the beads.

Initial experiments were with Protein A-coated large beads: this is a recombinant microbial protein that binds immunoglobulin at neutral pH; elution can only be achieved at pH <3 [33]. Protein A binding affinity can vary between antibody species and sub-class, however previous studies have demonstrated that Protein A binds murine IgG with high affinity [34] therefore it was not surprising that the murine IgG anti-rat CD90 bound to these Protein A coated beads. Efficient cell capture was still obtained when beads were loaded with an incredibly small quantity of antibody. CD90^+^ cell depletion was reported using flow cytometry, as the essential technique at the analytical stage to quantify cell capture on a population scale supported by fluorescent microscopy and qRT-PCR which identified CD90 transcript specifically associated with the beads. Collectively this cross referenced and validated this technique for isolation of CD90^+^ cells from heterogeneous SVF.

Although isolation of cells with Protein A beads was demonstrated to be of high efficiency an effective cellular release method was still required and so an alternative cell capture bead was explored for this purpose. This bead was modified with a covalently bound mixed-mode ligand coating based on an aromatic acid moiety; these ligands bind and release immunoglobins in a pH dependant manner over a narrow range and have been used in preparative chromatographic processes [26]. Otherwise, the overall structure and density of this mixed mode ligand bead was identical to the Protein A counterpart. This investigation successfully demonstrated loading of FITC-conjugated antibody onto mixed mode ligand beads at pH5-6, whilst raising the pH to 8.4 instantly released the antibody and subsequent bound cells. Pre-incubating ligand beads with an excess of polyclonal IgG (rabbit serum) prior to release significantly increased release efficiency. This suggested that saturating the ligand binding sites on the beads with non-specific IgG reduced the possibility of multiple interactions with the cell-specific antibody leading to optimal release kinetics.

This study therefore presents the initial steps in the validation of a new, minimally invasive stem cell harvesting system. Future research will focus on confirming and quantifying cell viability and phenotype maintenance in response to subjection to this novel pH mediated sorting strategy.

### Conclusion

A new approach to isolating highly purified populations of cells from primary complex mammalian tissues has been experimentally evidenced and validated. The technique marks a technical breakthrough in derivation of specific cell types based on high affinity selection. The cell isolation target molecule can be freely chosen on the basis of what is known about the cell type of choice. This approach provides a platform technology breakthrough that has been demonstrated here for adSCs but can by design and target molecule be tailored to any cell and tissue as required. It was not the authoring research teams intention to “invent” a new technique for cell isolation however limitations in the currently available techniques forced new approaches to be considered and developed to progress delivery of cell based therapies into man and animals and provide the most relevant *in vitro* 3D tissue analogues. The value and novelty in this approach lies in its non-invasive nature at a cellular level in addition to its ideal physical properties to be used with minimally processed primary tissue. The ability to tailor this approach by simply varying the antibody used makes its applications across cell biology and biomedicine vast. As demonstrated here this strategy may be used to exploit adipose as a clinical cell source with straight forward translation based on a cell population with an absolute minimum of exogenous manipulation prior to delivery.

## References

[1] Ohlstein B, Kai T, Decotto E, Spradling A (2004) The stem cell niche: theme and variations. Curr Op Cell Biol 6: 693–699. 10.1016/j.ceb.2004.09.003 15530783

[2] Mitsiadis TA, Barrandon O, Rochat A, Barrandon Y, De Bari C (2007) Stem cell niches in mammals. Exp Cell Res 313: 3377–3385. 17764674 10.1016/j.yexcr.2007.07.027

[3] Flake A (2004) The conceptual application of systems theory to stem cell biology: a matter of context. Blood Cell Mol Dis 32: 58–64. 10.1016/j.bcmd.2003.09.015 14757414

[4] Friedenstein AJ, Gorskaja JF, Kulagina NN (1976) Fibroblast precursors in normal and irradiated mouse hematopoietic organs. Exp Hematol 4: 267–274. 976387

[5] Dvorakova J, Hruba A, Velebny V, Kubala L (2008) Isolation and characterisation of mesenchymal stem cell populations entrapped in bone marrow collection sets. Cell Biol Int 32: 1116–1125. 18562221 10.1016/j.cellbi.2008.04.024

[6] Romanov YA, Darevskaya AN, Merlikina NV, Buravkova LB (2005) Mesenchymal stem cells from human bone marrow and adipose tissue: isolation, characterisation and differentiation potentialities. Bull Exp Biol Med 140: 138–143. 16254640 10.1007/s10517-005-0430-z

[7] Kruse C, Kajahn J, Petschnik AE, Maab A, Klink E, et al (2006) Adult pancreatic stem/progenitor cells spontaneously differentiate in vitro into multiple cell lineages and form teratoma-like structures. Annals of Anatomy 188: 503–506. 17140143 10.1016/j.aanat.2006.07.012

[8] Zuk PA, Zhu M, Mizuno H, Huang J, Futrell JW, et al (2001) Multilineage cells from human adipose tissue: implications for cell-based therapies. Tissue Eng 7: 211–228. 11304456 10.1089/107632701300062859

[9] Gronthos S, Mankani M, Brahim J, Robey PG, Shi S (2000) Postnatal human dental pulp stem cells (DPSCs) in vitro and in vivo. Proc Natl Acad Sci 97: 13625–13630. 11087820 10.1073/pnas.240309797 PMC17626

[10] Gronthos S, Brahim J, Li W, Fisher LW, Cherman N, et al (2002) Stem cell properties of human dental pulp stem cells. J Dent Res 81: 531–535. 12147742 10.1177/154405910208100806

[11] Huang AHC, Chen YK, Lin LM, Shieh TY, Chan AW (2008) Isolation and characterisation of dental pulp stem cells from a supernumerary tooth. J Oral Pathol Med 37: 571–574. 18331285 10.1111/j.1600-0714.2008.00654.x

[12] Qiao C, Xu W, Zhu W, Hu J, Qian H, et al (2008) Human mesenchymal stem cells isolated from the umbilical cord. Cell Biol Int 32: 8–15. 17904875 10.1016/j.cellbi.2007.08.002

[13] Kestendjieva S, Kyurkchiev D, Tsvetkova G, Mehandjiev T, Dimitrov A, et al (2008) Characterisation of mesenchymal stem cells isolated from the human umbilical cord. Cell Biol Int 32: 724–732. 18396423 10.1016/j.cellbi.2008.02.002

[14] Jiang Y, Jahagirdar BN, Reinhardt RL, Schwartz RE, Keene CD, et al (2002) Pluripotency of mesenchymal stem cells derived from adult marrow. Nature 418: 41–49. 12077603 10.1038/nature00870

[15] Banas A, Yamamoto Y, Teratani T, Ochiya T (2007) Stem cell plasticity: learning from hepatogeneic differentiation strategies. Dev Dynam 236: 3228–3241. 10.1002/dvdy.21330 17907200

[16] Arthur A, Rychkov G, Shi S, Koblar SA, Gronthos S (2008) Adult human dental pulp stem cells differentiate toward functionally active neurons under appropriate environmental cues. Stem Cells 26: 1787–1795. 18499892 10.1634/stemcells.2007-0979

[17] Pountos I, Corscadden D, Emery P, Giannoudis PV (2007) Mesenchymal stem cell tissue engineering: techniques for isolation, expansion and application. Injury 38: 23–33. 10.1016/s0020-1383(08)70006-8 18224734

[18] Zuk PA, Zhu M, Mizuno H, Huang J, Futrell JW, et al (2001) Multilineage cells from human adipose tissue: implications for cell-based therapies. Tissue Eng 7: 211–228. 11304456 10.1089/107632701300062859

[19] Zhu Y, Liu T, Song K, Fan X, Ma X, et al (2008) Adipose-derived stem cells: a better stem cell than BMSC. Cell Bioch Func 26: 664–675. 10.1002/cbf.1488 18636461

[20] Kern S, Eichler H, Stoeve J, Kluter H, Bieback K (2006) Comparative analysis of mesenchymal stem cells from bone marrow, umbilical cord, or adipose tissue. Stem Cells 24: 294–1301. 10.1634/stemcells.2005-0342 16410387

[21] Musina RA, Bekchanova ES, Sukhikh GT (2005) Comparison of mesenchymal stem cells isolated from different human tissues. Bull Exp Biol Med 139: 504–509. 16027890 10.1007/s10517-005-0331-1

[22] Rodbell M, Jones AB (1966) The metabolism of isolated fat cells. The similar inhibitory actions of phospholipase C (clostridium perfringens alpha toxin) and insulin on lipolysis stimulated by lipolytic hormones and theophylline. J Biol Chem 241: 140–142. 4285132

[23] Handley ME, Pollara G, Chain BM, Katz DR (2005) The use of targeted microbeads for quantitative analysis of the phagocytic properties of human monocyte-derived dendritic cells. J Immunol Meth 297: 27–38. 10.1016/j.jim.2004.11.009 15777928

[24] Mödder UI, Roforth MM, Nicks KM, Peterson JM, McCready LK, et al (2012) Characterization of mesenchymal progenitor cells isolated from human bone marrow by negative selection. Bone 3: 804–10. 10.1016/j.bone.2011.12.014 PMC3278574 22226689

[25] McKenzie KP, Mayer DC, Aubin JE (2012) Osteogenesis and expression of the bone marrow niche in endothelial cell-depleted HipOPs. J Cell Biochem. Epub ahead of print. 10.1002/jcb.24446 23161750

[26] Knudsen KL, Hansen MB, Henriksen LR, Andersen BK, Lihme A (1992) Sulphone aromatic ligands for theophilic adsorption chromatography: Purification of human and mouse immunoglobulins. Analytical Biochemistry 201: 170–177. 1621957 10.1016/0003-2697(92)90191-9

[27] Zhao G, Dong XY, Sun Y (2009) Ligands for mixed-mode protein chromatography: principles, characteristics and design. J Biotech 144: 3–11. 10.1016/j.jbiotec.2009.04.009 19409941

[28] Francesco FD, Tirino V, Desiderio V, Ferraro G, D'Andrea F, et al (2009) Human CD34^+^/CD90^+^ ASCs are capable of growing as sphere clusters, producing high levels of VEGF and forming capillaries. PLoS ONE 4: e6537. 19657392 10.1371/journal.pone.0006537 PMC2717331

[29] Ishimura D, Yamamoto N, Tajima K, Ohno A, Yamamoto Y, et al (2008) Differentiation of adipose-derived stromal vascular fraction culture cells into chondrocytes using the method of cell sorting with a mesenchymal stem cell marker. Tohoku J Exp Med 216: 149–156. 18832797 10.1620/tjem.216.149

[30] Willoughby N (2009) To big to bind? Will the purification of large and complex therapeutic targets spell the beginning of the end for column chromatography. J Chem Technol Biot 84: 145–150.

[31] Balmayor ER, Pashkuleva L, Frias AM, Azevedo HS, Reis RL (2011) Synthesis and functionalization of superparamagnetic poly-{varepsilon}-caprolactone microparticles for the selective isolation of subpopulations of human adipose-derived stem cells. J R Soc Interface 8: 896–908. 21208971 10.1098/rsif.2010.0531 PMC3104349

[32] Ujam LB, Clemmitt RH, Clarke SA, Brooks RA, Rushton N, et al (2003) Isolation of monocytes from human from human peripheral blood using immune-affinity expanded bed adsorption. Biotechnol Bioeng 83: 554–566. 12827697 10.1002/bit.10703

[33] Lindmark R, Thorén-Tolling K, Sjöquist J (1983) Binding of immunoglobulins to protein A and immunoglobulin levels in mammalian sera. J Immunol Meth 62: 1–13. 10.1016/0022-1759(83)90104-7 6348168

[34] Ey PL, Prowse SJ, Jenkin CR (1978) Isolation of pure IgG1, IgG2a, and IgG2b immunoglobulins from mouse serum using protein A-sepharose. Immunochemistry 15: 429–436. 30693 10.1016/0161-5890(78)90070-6

